# Temporal Gene Expression Profiles after Focal Cerebral Ischemia in Mice

**DOI:** 10.14336/AD.2017.0424

**Published:** 2018-04-01

**Authors:** Chengjie Zhang, Yanbing Zhu, Song Wang, Zheng Zachory Wei, Michael Qize Jiang, Yongbo Zhang, Yuhualei Pan, Shaoxin Tao, Jimei Li, Ling Wei

**Affiliations:** ^1^Department of Neurology, Beijing Friendship Hospital, Capital Medical University, Beijing 100050, China.; ^2^Laboratories of Stem Cell Biology and Neural Regeneration and Function Recovery, Beijing Friendship Hospital, Capital Medical University, Beijing 100050, China.; ^3^Department of Anesthesiology, Emory University School of Medicine, Atlanta, GA 30322, USA.

**Keywords:** experimental cerebral ischemia, RNA-seq, differentially expressed genes, inflammation related genes

## Abstract

A cascade of pathological processes is triggered in the lesion area after ischemic stroke. Unfortunately, our understanding of these complicated molecular events is incomplete. In this investigation, we sought to better understand the detailed molecular and inflammatory events occurring after ischemic stroke. RNA-seq technology was used to identify whole gene expression profiles at days (D1, D3, D7, D14, D21) after focal cerebral ischemia in mice. Enrichment analyses based on Gene Ontology (GO) and Kyoto Encyclopedia of Genes and Genomes (KEGG) terms for the differentially expressed genes (DEGs) were then analyzed. Inflammation-related genes that were significantly expressed after stroke were selected for analysis and the temporal expression patterns of pro-inflammatory and anti-inflammatory genes were reported. These data illustrated that the number of DEGs increased accumulatively after cerebral ischemia. In summary, there were 1967 DEGs at D1, 2280 DEGs at D3, 2631 DEGs at D7, 5516 DEGs at D14 and 7093 DEGs at D21. The significantly enriched GO terms also increased. 58 GO terms and 18 KEGG pathways were significantly enriched at all inspected time points. We identified 87 DEGs which were functionally related to inflammatory responses. The expression levels of pro-inflammation related genes CD16, CD32, CD86, CD11b, Tumour necrosis factor α (TNF-α), Interleukin 1β (IL-1β) increased over time and peaked at D14. Anti-inflammation related genes Arginase 1 (Arg1) and Chitinase-like 3 (Ym1) peaked at D1 while IL-10, Transforming growth factor β (TGF-β) and CD206, which were induced at 1 day after cerebral ischemia, peaked by 7 to 14 days. These gene profile changes were potentially linked to microglia/macrophage phenotype changes and could play a role in astroglial activation. This study supplies new insights and detailed information on the molecular events and pathological mechanisms that occur after experimental ischemic stroke.

Ischemic stroke remains one of the leading causes of death in the world [1]. Once the cerebral arteries are blocked, a cascade of pathological processes are triggered in the ischemic lesion areas. These include increased Ca^2+^ that causes excitotoxicity, necrotic or apoptotic cell death, and activation and migration of inflammatory cells within the brain and from blood to the ischemic area [2-4]. Although the pathological mechanisms of ischemic stroke have been partly elucidated, our understanding of the detailed molecular events remains incomplete.

Several studies have found that inflammation is not just a reaction to ischemic tissue but that it played a key role in the pathophysiology of ischemic stroke [3, 5]. The inflammatory response is activated upon vessel occlusion, and it plays a role in all further stages of ischemic stroke. Various immune cells contribute to the inflammatory response and these cells can express inflammation-related proteins on their cell surface or release inflammatory factors into the extracellular environment [6-9]. However, the inflammatory response does not only accelerate the damage of ischemic tissue, it may also play a role in repairing the ischemic tissue.

RNA-seq technology has been used to understand pathological mechanisms in nervous system diseases, such as stroke, Alzheimer’s disease, spinal cord disease [10-12]. In this study, we employed RNA-seq technology to identify the whole gene expression profiles at different time points after experimental cerebral ischemia. The DEGs, the GO enrichment analysis and the KEGG pathway analyses were used to investigate the pathological mechanisms of ischemic stroke. In order to better understand the inflammatory response, related DEGs were selected and temporal expression levels of pro-inflammation and anti-inflammation related genes were determined after focal cerebral ischemia in mice.

## MATERIALS AND METHODS

### Animal model

Adult male mice aged 8-10 weeks were purchased from Beijing Vital River Laboratory Animal Technology Co. Ltd., (Beijing, China). All mice were housed under a 12 h light/dark cycle at room temperature (23°C) in the pathogen-free laboratory animal center of Beijing Friendship Hospital, Capital Medical University (Beijing, China). All experiments were performed according to protocols approved by the Animal Studies Subcommittee of the Capital Medical University.

There were 6 groups: sham control mice, 1 day (D1), 3 days (D3), 7 days (D7), 14 days (D14) and 21 days (D21) after cerebral ischemic stroke (n=6, pooled 3 mice together each time and had a biological replicate). Two mice were used for TTC staining at D1 after ischemic stroke. To induce the focal cerebral ischemic stroke, we ligated the distal middle cerebral artery (MCA) and temporarily (7 min) occluded both common carotid arteries (CCAs), as described previously [13]. Briefly, mice were anesthetized with 10% chloral hydrate (100 mg/kg, i. p.). A 7-mm skin incision was made between the orbit and the ear on the right side. After separating the muscle, a 3 mm diameter hole was drilled with a dental drill to expose the MCA. Distal branches of MCA were permanently ligated with 10 sterile sutures accompanied by a temporary (7 min) occlusion of both common carotid arteries (CCAs). Finally, the muscle and skin incision was sutured. Sham surgeries were identical to stroke surgeries without ligating MCA branches. The body temperature of the animals was maintained at 37 ± 0.5 °C during the surgery and up to 2 hours after surgery.

### Tissue preparation

Brain tissue was obtained from the ischemic lesion area which included core and penumbra at D1, D3, D7, D14 and D21 after cerebral ischemia. The same area in sham control mice brain was also obtained. The animals were weighed, deeply anesthetized, and perfused with 25 ml of saline phosphate buffer (PBS: 0.1 M, pH 7.3) before we obtained the brain tissue. TTC (2,3,5-triphenyltetrazolium chloride) staining was used to show the ischemic lesion area of the brain tissue at D1. The brains were sliced into 1 mm slices by using a coronal brain matrix. The sections were placed into 2% TTC at 37°C for 5-10?min and then the sections were stored in 4% buffered paraformaldehyde for scanning.

### RNA-sequencing

RNA-seq was performed on the RNA isolated from brain tissue. A total of 3 μg RNA of each group was used for the sample preparations. The NEBNext® UltraTM RNA Library Prep Kit for Illumina® (NEB, USA) was used to obtain the sequencing libraries. The TruSeq PE Cluster Kit v3-cBot-HS (Illumia) was used to cluster the index-coded samples. After cluster generation, the samples were sequenced via Illumina Hiseq platform.

### Data analysis

In order to obtain clean reads, the reads which contained the adapter, poly-N, and low-quality reads were removed. The clean reads were mapped to the reference genome via TopHat v2.0.12 and the read number that mapped to the genes via HTSeq v0.6.1 was counted. The FPKM value was used to estimate the gene expression levels. The DEGs analysis among the D1 vs Sham, D3 vs Sham, D7 vs Sham, D14 vs Sham and D21 vs Sham were performed by using DESeq R package (1.18.0). The pval < 0.05 and |log2(Foldchange)| >= 0?were assigned as statistically significant differences. The GO enrichment analysis of DEGs was performed by the GOseq R package. The KEGG pathway analysis was performed by KOBAS software. The pval < 0.05 was assigned as a statistically significant difference.

### Inflammatory response related genes

The inflammatory response related genes which were significantly expressed at all the time points were selected. These included cytokines and related receptors, complement and related receptors, surface antigens, C-type lectin family and major histocompatibility complex. The temporal expression levels (FPKM value) of pro-inflammatory (CD16, CD32, CD86, CD11b, TNF-α and IL-1β) and anti-inflammatory (CD206, Arg1, Ym1, IL-10 and TGF-β) related genes were also estimated after cerebral ischemia.

### Quantitative real-time PCR validation

Quantitative real-time PCR (qRT-PCR) analysis of 6 inflammatory response related genes (CD32, CD86, CD206, Arg1, Ym1, TGF-β) were performed to confirm temporal expression levels in the RNA-seq results. The primers used in the qRT-PCR are listed in (Table 1). A qRT-PCR was performed on the Applied Biosystems 7500 Fast Real-Time PCR System using PowerUp SYBR Green Master Mix (Thermo Fisher Scientific). β-actin was used as the normalization control. The relative quantification of the target genes was calculated by using the 2^-ΔΔ(Ct)^ method.

**Table 1:** The primers for qRT-PCR.

Gene name	Primer	Sequence (5’–3’)
CD32	Forward	AGGGCCTCCATCTGGACTG
	Reverse	GTGGTTCTGGTAATCATGCTCTG
CD86	Forward	GGTGGCCTTTTTGACACTCTC
	Reverse	TGAGGTAGAGGTAGGAGGATCTT
CD206	Forward	GAGGGAAGCGAGAGATTATGGA
	Reverse	GCCTGATGCCAGGTTAAAGCA
Arg1	Forward	CTCCAAGCCAAAGTCCTTAGAG
	Reverse	AGGAGCTGTCATTAGGGACATC
Ym-1	Forward	AGGACTCCTGGCTTACTATGA
	Reverse	AACCAACCCACTCATTACCC
TGF β	Forward	TCTGCATTGCACTTATGCTGA
	Reverse	AAAGGGCGATCTAGTGATGGA
beta-actin	Forward	GGCTGTATTCCCCTCCATCG
	Reverse	CCAGTTGGTAACAATGCCATGT

### Immunofluorescence staining

Mice were weighed, deeply anesthetized, and transcardially perfused with PBS, followed by 4% paraformaldehyde (PFA) at each time point. Brains were carefully dissected, postfixed overnight, cryoprotected in 30% sucrose solution, and embedded in tissue-freezing medium for cryostat sectioning. Frozen brains were cut into 10 μm thick coronal cryostat sections. The sections were incubated with the primary antibodies (mouse anti-NeuN, 1:200, Millipore; rat anti-IBA1, 1:200, Bio-Rad Laboratories; rabbit anti-GFAP, 1:200, Millipore) overnight at 4°C. Sections were washed with PBS for 3 times and incubated with the secondary antibody (Alexa Fluor 488 anti-rat or rabbit, 1:400, Abcam; Cy3 conjugated anti-mouse, 1:400, Jackson ImmunoResearch Laboratories) for 1 h at room temperature. After washing 3 times with PBS, the nuclei were stained with Hoechst 33342 (1:20000, Life Technologies) for 5 min.

## RESULTS

### Temporal genome-wide expression after ischemic stroke.

**Figure 1.**
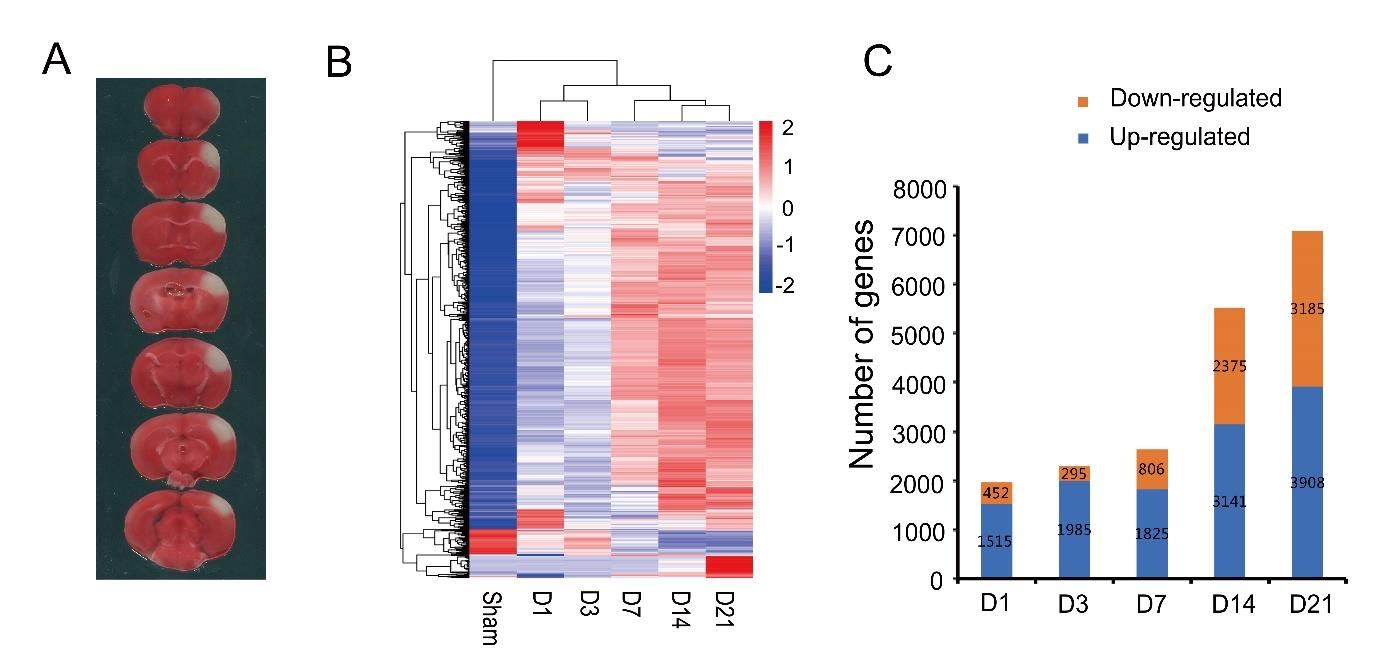
TTC staining and differentially expressed genes after cerebral ischemia. A) TTC staining of brain at D1 after ischemic stroke showing the ischemic lesion area in the cortex (white). B) Heatmap of the up-regulated and down-regulated DEGs between the post-stroke groups and sham group. C) The number of upregulated and downregulated DEGs at different time points. Increase in the total DEG numbers were observed over time from Day 7 to Day 21 after stroke.

TTC staining was used to show the ischemic lesion area in the cortex (in white) (Fig. 1A). In order to explain the molecular mechanisms after ischemic stroke, the changes of gene expression in the ischemic lesion area were analyzed. The temporal gene expression profiles at five post-stroke time points: D1, D3, D7, D14 and D21 were compared to the sham group (Fig. 1B). There were 1967 DEGs at D1 with 1515 up-regulated and 452 down-regulated, 2280 DEGs at D3 with 1985 up-regulated and 295 down-regulated, 2631 DEGs at D7 with 1825 up-regulated and 806 down-regulated, 5516 DEGs at D14 with 3141 up-regulated and 2375 down-regulated, 7093 DEGs at D21 with 3908 up-regulated and 3185 down-regulated (Fig. 1C). The number of DEGs gradually increased, with more up-regulated genes than down-regulated genes at all time points. The top 10 highest DEGs for different time points were summarized (Table 2).

### Gene ontology enrichment analysis of the DEGs at different time points.

As for the up-regulated and down-regulated genes in the ischemic lesion area of the brain, the DEGs were analyzed based on GO terms of biological process, cellular component and molecular function.

**Figure 2.**
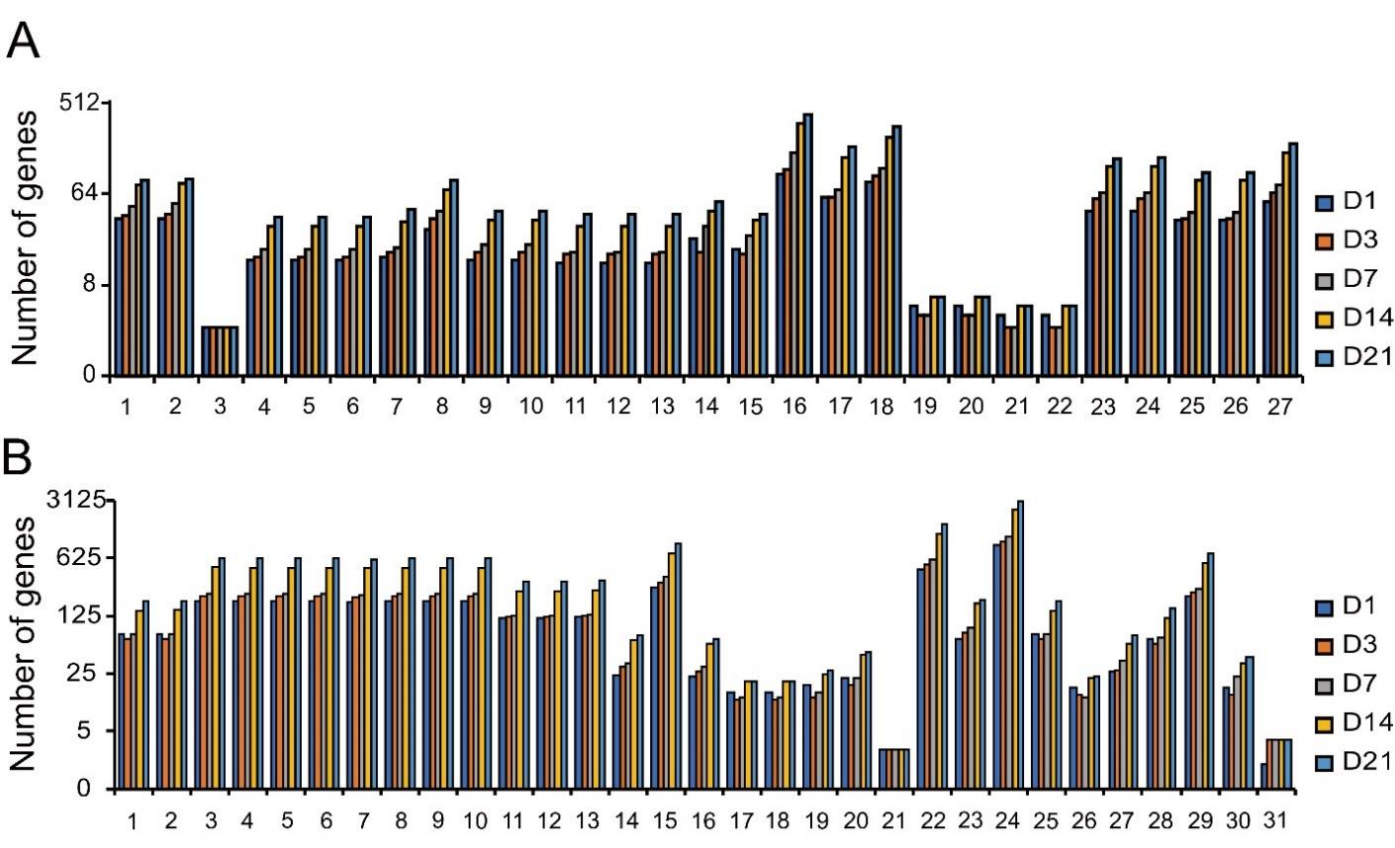
Gene ontology enrichment analysis of the DEGs at all the time points after cerebral ischemia. A) 27 significant terms of biological process were enriched at all the inspected time points and the number of DEGs in the terms were increased (1.immune response 2.immune system process 3.production of molecular mediator involved in inflammatory response 4.regulation of cell death 5.regulation of apoptotic process 6.regulation of programmed cell death 7.regulation of autophagy 8.autophagy 9.apoptotic process 10.programmed cell death 11.positive regulation of autophagy 12.positive regulation of catabolic process 13.positive regulation of cellular catabolic process 14.divalent metal ion transport 15.iron ion transport 16.ion transport 17.small GTPase mediated signal transduction 18.intracellular signal transduction 19.regulation of microtubule-based process 20.regulation of microtubule cytoskeleton organization 21.microtubule polymerization or depolymerization 22.regulation of microtubule polymerization or depolymerization 23.cell adhesion 24.biological adhesion 25.protein complex assembly 26.protein complex biogenesis 27.protein phosphorylation). B) 31 significant terms in molecular function were enriched at all the different time points and the number of DEGs in these terms were increased (1. guanyl ribonucleotide binding 2. guanyl nucleotide binding 3. purine nucleotide binding 4. purine nucleoside binding 5. ribonucleoside binding 6. purine ribonucleoside binding 7. purine ribonucleoside triphosphate binding 8. purine ribonucleotide binding 9. ribonucleotide binding 10. nucleoside binding 11. nucleoside-triphosphatase activity 12. pyrophosphatase activity 13. hydrolase activity, acting on acid anhydrides, in phosphorus-containing anhydrides 14. hydrolase activity, acting on glycosyl bonds 15. hydrolase activity 16. hydrolase activity, hydrolyzing O-glycosyl compounds 17. chemokine activity 18. chemokine receptor binding 19. cytokine activity 20. cytokine receptor binding 21. cytokine receptor activity 22. protein binding 23. calcium ion binding 24. binding 25. GTP binding 26. G-protein coupled receptor binding 27. lipid binding 28. GTPase activity 29. anion binding 30. iron ion transmembrane transporter activity 31. glutathione peroxidase activity).

Regarding biological processes, there were 149 significant terms for D1T-shamT, 129 significant terms for D3T-shamT, 158 significant terms for D7T-shamT, 186 significant terms for D14T-shamT, 180 significant terms for D21T-shamT. 27 significant terms were enriched at all the different time points and the number of DEGs in the terms were gradually increased (Fig. 2A). Immune response, inflammatory response, cell death, apoptotic, autophagy, and ion transport were the most enriched terms of biological process at different time points after ischemic stroke.

Regarding cellular components, there were 30 significant terms for D1T-shamT, 31 significant terms for D3T-shamT, 46 significant terms for D7T-shamT, 61 significant terms for D14T-shamT, 57 significant terms for D21T-shamT. There were no same enriched clusters of cellular components at any of the time points.

Regarding molecular functions, there were 103 significant terms for D1T-shamT, 102 significant terms for D3T-shamT, 121 significant terms for D7T-shamT, 140 significant terms for D14T-shamT, 138 significant terms for D21T-shamT. 31 significant terms were enriched at all the different time points and the number of DEGs in the terms were also gradually increased (Fig. 2B). The most enriched terms included: protein binding, calcium ion binding, guanyl ribonucleotide binding, chemokine activity, and chemokine receptor binding at all time points.

**Table 2:** The top 10 highest DEGs at different time points.

	Gene Name	log2Fold Change	pval	Description
**D1**	Mmp3	9.095	6.55E-16	matrix metallopeptidase 3
	Il11	8.409	1.02E-05	interleukin 11
	Ccl4	7.786	5.06E-11	chemokine (C-C motif) ligand 4
	Ccl2	7.7254	1.65E-33	chemokine (C-C motif) ligand 2
	Arg1	7.5703	7.02E-20	arginase
	Il6	7.1652	2.31E-14	interleukin 6
	Tfpi2	6.9083	4.82E-07	tissue factor pathway inhibitor 2
	Clec4e	6.8167	0.0003859	C-type lectin domain family 4, member e
	Ptx3	6.7667	2.63E-35	pentraxin related gene
	Ccl12	6.7128	1.92E-49	chemokine (C-C motif) ligand 12
**D3**	Apoc2	8.2917	5.43E-06	apolipoprotein C-II
	Nxpe5	7.8031	1.87E-07	neurexophilin and PC-esterase domain family, member 5
	Aplnr	7.2269	1.86E-12	apelin receptor
	Cd5l	7.1734	5.37E-06	CD5 antigen-like
	Tfpi2	6.9459	0.0046113	tissue factor pathway inhibitor 2
	Arg1	6.8394	0.0031864	arginase
	Pyhin1	6.7744	9.15E-05	pyrin and HIN domain family, member 1
	Bub1	6.7704	4.73E-08	budding uninhibited by benzimidazoles 1 homolog
	Cdk1	6.5985	4.27E-13	cyclin-dependent kinase 1
	Msr1	6.4753	0.0015781	macrophage scavenger receptor 1
**D7**	Apoc2	9.9106	5.20E-06	apolipoprotein C-II
	Gpnmb	9.8564	0.0072568	glycoprotein (transmembrane) nmb
	Spp1	9.842	0.0091977	secreted phosphoprotein 1
	Cd5l	9.7619	0.00099001	CD5 antigen-like
	Mmp3	9.4058	0.018527	matrix metallopeptidase 3
	H19	9.2454	0.0029637	H19, imprinted maternally expressed transcript
	Mmp13	9.0637	0.017491	matrix metallopeptidase 13
	Clec7a	8.9704	0.0010311	C-type lectin domain family 7, member a
	Mcoln3	8.85	0.014833	mucolipin 3
	Lgals3	8.8209	0.0018961	lectin, galactose binding, soluble 3
**D14**	Mmp3	12.888	9.44E-22	matrix metallopeptidase 3
	Saa3	12.007	1.24E-49	serum amyloid A 3
	Oas3	10.535	2.03E-07	2’-5’ oligoadenylate synthetase 3
	Pyhin1	10.529	1.01E-05	pyrin and HIN domain family, member 1
	Mmp13	10.421	2.20E-25	matrix metallopeptidase 13
	Apoc2	10.186	1.58E-30	apolipoprotein C-II
	Cst7	10.095	3.66E-20	cystatin F (leukocystatin)
	Clec7a	9.8254	7.85E-09	C-type lectin domain family 7, member a
	Ccl5	9.8228	9.61E-28	chemokine (C-C motif) ligand 5
	Mmp12	9.4518	7.62E-35	matrix metallopeptidase 12
**D21**	Igha	13.067	1.41E-92	immunoglobulin heavy constant alpha
	Mmp3	12.209	1.35E-78	matrix metallopeptidase 3
	Cd5l	11.764	1.43E-13	CD5 antigen-like
	Saa3	10.794	2.78E-28	serum amyloid A 3
	C3	10.276	8.25E-29	complement component 3
	Clec7a	10.228	1.63E-17	C-type lectin domain family 7, member a
	Igkc	10.113	3.13E-85	immunoglobulin kappa constant
	Ccl5	10.108	3.02E-43	chemokine (C-C motif) ligand 5
	Mmp12	10.082	3.63E-08	matrix metallopeptidase 12
	Cxcl13	9.825	2.95E-07	chemokine (C-X-C motif) ligand 13

### The pathway analysis of differentially expressed genes at different time points.

To further analyze the function of DEGs in the ischemic lesion area of the brain at all the time points, the KEGG pathway analysis was used. There were 47 significant pathways for D1T-shamT, 47 significant pathways for D3T-shamT, 45 significant pathways for D7T-shamT, 53 significant pathways for D14T-shamT, 48 significant pathways for D21T-shamT. At the same time, the pathways which were significantly clustered at all the time points were selected and divided into two groups: function-related and disease-related (Table 3). The function-related pathways included: TNF signaling pathway, phagosome, ECM-receptor interaction, platelet activation, Fc gamma R-mediated phagocytosis, NOD-like receptor signaling pathway, B cell receptor signaling pathway, cell adhesion molecules (CAMs) and osteoclast differentiation. The disease-related pathways were involved in: Pertussis, Leishmaniasis, Staphylococcus aureus infection, Tuberculosis, Chagas disease (American trypanosomiasis), Rheumatoid arthritis, Toxoplasmosis, Amoebiasis and Inflammatory bowel disease (IBD) based on KEGG.

**Figure 3.**
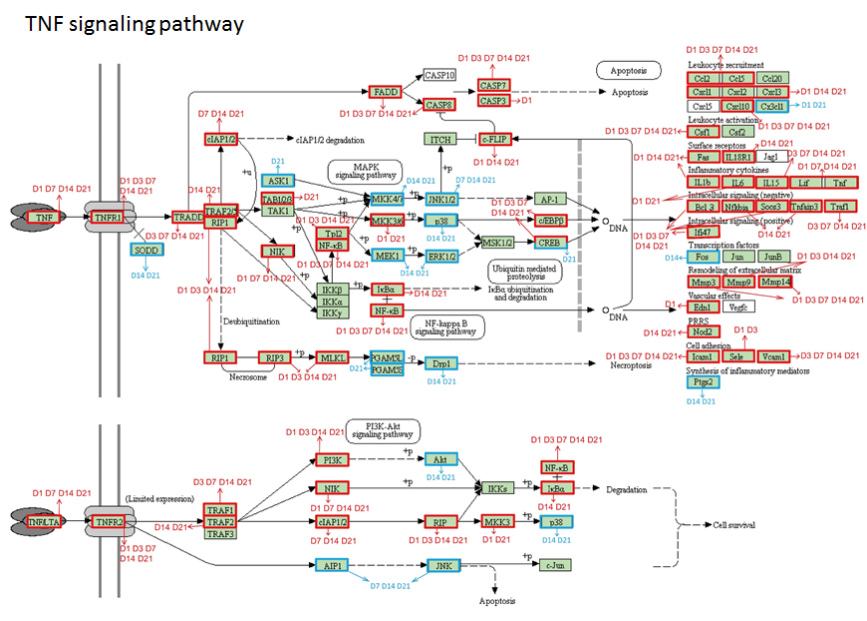
The DEGs associated with TNF signaling pathway at different time points after cerebral ischemia. The upregulated genes are boxed in red and the down-regulated genes in blue. Arrows indicate the time points of up-regulated genes (red) and down-regulated genes (blue).

The TNF signaling pathway is one of the most enriched KEGG pathways. Here, its function encompasses three biological processes: apoptosis, inflammation/immunity and cell survival. Therefore, the change of genes in this pathway are observed at varying time points. 40 genes were up regulated and 1 gene was down regulated at D1; 30 genes were up regulated at D3; 29 genes were up regulated and 2 genes were down regulated at D7; 48 genes were up regulated and 13 genes were down regulated at D14; 51 genes were up regulated and 16 genes were down regulated at D21 (Fig. 3). Apoptosis related genes such as Fas-associated via death domain (Fadd), Caspase8, Caspase7, Caspase3 were up regulated even at D21 after ischemic stroke. Adhesion molecules, including intercellular adhesion molecule 1 (Icam1), Selectin (Sele), and vascular cell adhesion molecule 1 (Vcam1), were also highly expressed according to our RNA-seq results. Besides, many chemokines in this pathway, including Ccl2, Cxcl1, Cxcl2, Cxcl3 and Cxcl10, were also significantly upregulated. IL-1β, IL-6, IL-15, leukemia inhibitory factor (LIF), TNF-α were the significantly expressed inflammatory cytokines. LIF was up regulated at D1 (with the log2Fold change at D1, D3, D7, D14, D21: 5.1705, 1.9495, 2.2962, 2.0899, 1.9872) and then gradually decreased with the progression of ischemic stroke. This may imply a role of LIF in the early period after ischemic stroke.

**Table 3:** The KEGG pathways significantly enriched at all the time points.

Description	Input number of genes					Total number
	D1	D3	D7	D14	D21	
Function related pathways						
TNF signaling pathway	41	30	31	61	67	**109**
Phagosome	52	53	56	85	98	**174**
ECM-receptor interaction	32	34	24	49	54	**88**
Platelet activation	40	41	36	66	79	**131**
Fc gamma R-mediated phagocytosis	27	26	25	51	60	**88**
NOD-like receptor signaling pathway	20	16	20	35	39	**58**
B cell receptor signaling pathway	20	23	22	42	50	**73**
Cell adhesion molecules (CAMs)	41	41	53	78	91	**160**
Osteoclast differentiation	45	43	43	73	79	**126**
Disease related pathways						
Pertussis	31	27	27	48	51	**74**
Leishmaniasis	28	25	27	42	45	**65**
Staphylococcus aureus infection	24	27	28	35	34	**51**
Tuberculosis	53	51	52	89	106	**176**
Chagas disease (American trypanosomiasis)	35	28	27	59	71	**103**
Rheumatoid arthritis	30	24	28	44	57	**82**
Toxoplasmosis	36	29	36	54	68	**113**
Amoebiasis	37	32	31	58	69	**119**
Inflammatory bowel disease (IBD)	21	19	22	33	38	**59**

### Inflammatory response related genes

Inflammatory responses have been implicated in the pathological mechanisms of ischemic stroke. 87 inflammation-related genes that are significantly expressed at all the time points (which included cytokine and receptors, complement and receptors, surface antigens, C-type lectin family and major histocompatibility complex) were selected for analysis (Table 4). The data supported that a variety of related genes and their expression patterns could be involved in regulating post-stroke inflammatory responses.

**Figure 4.**
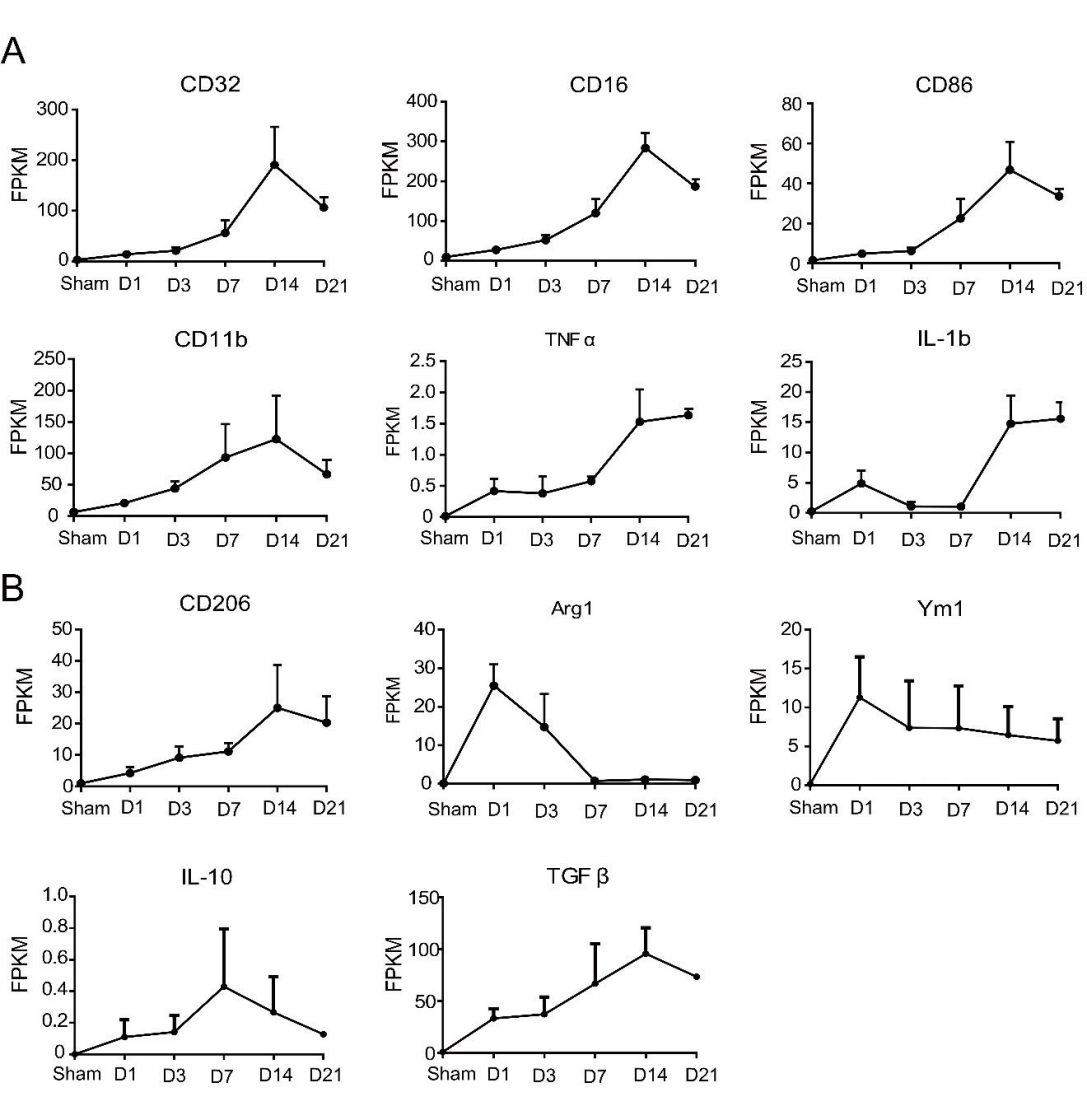
Expression levels (FPKM value) of the pro-inflammatory and anti-inflammation related genes that were significantly expressed after cerebral ischemia. A) The pro-inflammatory related genes (CD16, CD32, CD86, CD11b, TNF-a and IL-1b); B) The anti-inflammation related genes (CD206, Arg1, Ym1, IL-10 and TGF β).

Next, a total of 11 microglia/microphage and astrocyte related cytokines/markers were summarized among the 87 inflammation-related genes. Expression levels (FPKM values) of CD16, CD32, CD86 and CD11b were increased at D1 and peaked at D14 after ischemic stroke. The level of TNF-α was gradually increased at D1 with a flat period from D14 to D21. The level of IL-1β was not very high from D1 to D7, but peaked at D14 followed by a flat period from D14 to D21 (Fig. 4A). In contrast to the pro-inflammatory related genes, expression levels of Arg1 and Ym1 peaked at D1 and decreased over time, whereas expression levels of IL-10, TGF-β and CD206 peaked at the later time points (IL-10 at D7, TGF-β and CD206 at D14) (Fig. 4B). These results indicated that the peak time for expression of pro-inflammation related genes is later than the anti-inflammatory related genes.

### Identification of the temporal expression levels of inflammatory response related genes

**Figure 5.**
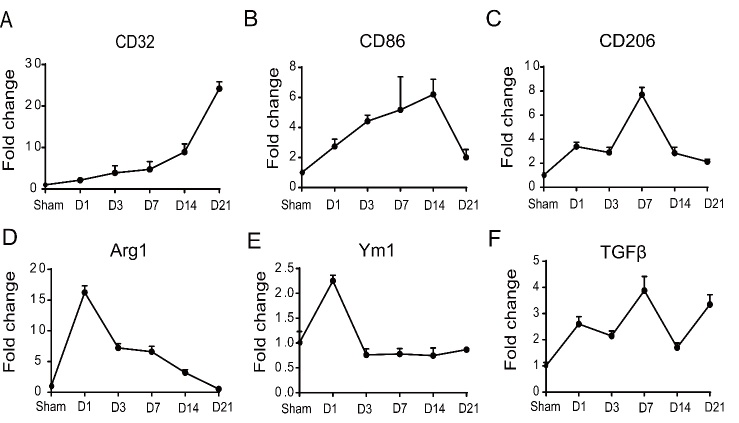
Identification of the temporal expressions of inflammatory response related genes with qRT-PCR. The expression level of CD32 (A) continuously increased and remained elevated at D21 after ischemic stroke. The level of CD86 (B) gradually increased at D1 and peaked at D14 after ischemic stroke. Expression levels of CD206 (C) and TGF-β (F) peaked at D7. Levels of Arg1 (D) and Ym1 (E) peaked at D1.

Among the 11 identified temporally regulated inflammatory response-related genes, six (CD32, CD86, CD206, Arg1, Ym1, TGF-β) were validated by qRT-PCR (Fig. 5). An expression level of CD32 was gradually increased and remained elevated at D21 after ischemic stroke. CD86 was increased at D1 and peaked at D14 after ischemic stroke. Levels of TGF-β and CD206 peaked at D7. Arg1 and Ym1 peaked at D1, then were gradually decreased at later time points. Together, the temporal changes of those related genes were consistent with our RNA-seq results.

### Microglia/macrophages and astrocytes in peri-infarct regions after focal ischemia

**Figure 6.**
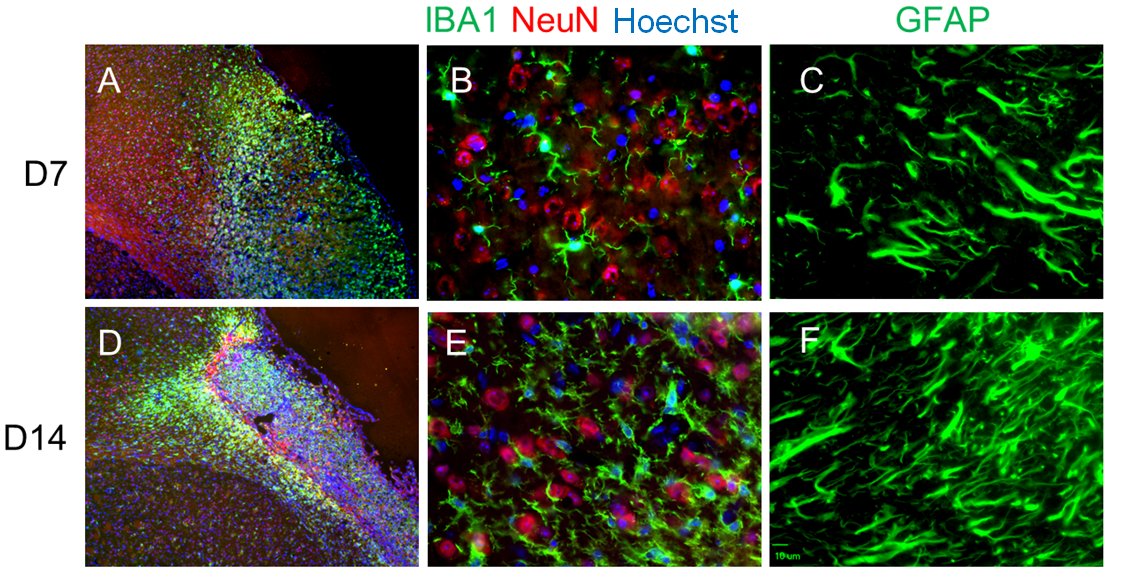
Microglia/macrophages and astrocytes in peri-infarct regions. Iba-1 (Green), NeuN (Red), and Hoechst (Blue) immunostaining at 7 days post ischemic stroke (A-C) featuring primarily ramified microglia. At a later time point of D14 (D-F), microglia displayed a more phagocytic phenotype. The level of GFAP also steadily increased over time starting from D1. GFAP staining shown at D7 (C) and D14 (F).

Ischemic lesion area was investigated by immunostaining Iba-1 and GFAP for microglia/macrophages and astrocytes. D14 and D21 were chosen based on inflammatory/anti-inflammatory gene expression profiles. There were many Iba-1-positive cells in the peri-infarct regions at D14 and D21 (Fig. 6A-C). Significant morphological changes of the microglia/macrophages were observed in this study. The data supported the anti-inflammatory changes of D14 and D21 microglia/macrophages, which were ramified compared to an amoeba phenotype (data not shown). Similarly, reactive astrocytes may be observed in the peri-infarct regions. Increased immunofluorescence was observed at D7 and D14 respectively alongside morphological changes in astrocyte endfeet. By D7, reactive GFAP positive astrocytes exhibit hypertrophied processes consistent with the expression profile patterns.

**Table 4:** The inflammatory related genes significantly expressed at all the time points.

Category	Gene symbols
Cytokine and receptors	Ifngr1 IL-10rb Il13ra1 Il17rc Il18rap Il21r Il2rg Il33 Il4ra Tnfaip2 Tnfrsf10b Tnfrsf13b Tnfrsf1a Tnfrsf1b Tnfrsf26 Csf1 Csf2rb Csf2rb2 Ccl12 Ccl3 Ccl4 Ccl5 Ccl6 Ccl7 Ccl9 Ccr1 Ccr2 Ccr5 Cmklr1 Cx3cr1 Cxcl10 Cxcl16
Complement and receptors	C1qa C1qb C1qc C1rl C3 C3ar1 C4b C5ar1 C5ar2
Surface antigens	Cd14 Cd151 Cd180 Cd248 Cd300lb Cd300ld Cd300lf Cd36 Cd37 Cd38 Cd44 Cd48 Cd52 Cd53 Cd5l Cd63 Cd68 Cd72 Cd74 Cd82 Cd84 Cd86 Cd9 Itgax Itgb2 Itgb7 Scarf1 Scarf2 Tlr1 Tlr13 Tlr2
C-type lectin family	Clec14a Clec2d Clec4a1 Clec4a2 Clec4n Clec7a
Major histocompatibility complex	H2-Aa H2-Ab1 H2-D1 H2-DMb1 H2-Eb1 H2-K1 H2-Q4 H2-Q5 H2-Q6

## DISCUSSION

In this study, RNA-seq technology was used to systematically characterize the temporal gene expression profiles of genes involved in the pathophysiology of cerebral ischemia in mice. Tissue from the ischemic lesion area of brain was obtained for RNA-seq based on TTC staining which demarcates the ischemic lesion by color. In order to better understand the pathological mechanisms and detailed molecular events, five time points (1 day, 3 days, 7 days, 14 days, and 21 days) after ischemic stroke were chosen for analysis and differentially expressed genes were identified and analyzed accordingly. The focal ischemic stroke model tested in this investigation is induced by permanent MCA occlusion plus transient CCA ligations, which resembles partial reperfusion injury commonly seen in stroke patients. As an intermediate model between permanent ischemia and full reperfusion after transient ischemia, we propose that the gene profile identified in this model is representative to some extent for both permanent and transient ischemic strokes.

Based on our bioinformatics methods, we observed many enriched up-regulated genes following ischemic stroke. Similarly, a previous study showed via microarray analysis that the number of DEGs at 3 days after experimental stroke was greater than at 24 hours [14]. An increasing number of DEGs with the progression of ischemic stroke were reported and there were more up-regulated genes than down-regulated genes.

Our results were consistent with many other reports of gene regulation after ischemia including effects observed in matrix metalloproteinases (MMPs), cytokines, and chemokines. MMPs, released by neutrophils and astrocytes, are able to destabilize the blood brain barrier after ischemic stroke [15, 16]. RNA-seq revealed significant MMP-3 up-regulation (highly expressed up to 21 days after cerebral ischemia). MMP-3 has been reported [14] to promote hemorrhagic transformation after ischemic stroke. In addition, many enriched inflammatory markers after cerebral ischemia, including interleukins IL-11 and IL-6, chemokines (Ccl4, Ccl2, Ccl5) and serum amyloid A (Saa3), were observed to be up-regulated consistent with reports elsewhere [17, 18]. We report 58 GO terms and 18 KEGG pathways were significantly enriched among all time points. Considering the cumulative increases in both the number of DEGs and the number of enriched GO and KEGG terms at each time point examined following ischemic stroke, we concluded that pathological processes post-ischemic stroke becomes increasingly complex over time. Evidence suggests that the immune system is involved at all stages following ischemic stroke and that continuous inflammatory responses occur throughout the pathophysiology of ischemic stroke. We thus focused on inflammation/cell death-related genes and their terms. In particular, TNF signaling pathway was one of the most enriched pathways. Apoptosis related genes (Fadd, Casp8, Casp7 and Casp3) were upregulated even at D21 [3, 19]. A previous study showed that the Fadd inhibitor decreased cell death after ischemic stroke [20]. Adhesion molecules that could promote leukocyte recruitment, adhesion to the endothelial surface and transmigration to the ischemic lesion area [5, 21] were found to be highly expressed by our analysis. An inhibition of Icam1 had protective effects after ischemic stroke as previously reported in a rat model [22]. We also noticed that LIF peaked at D1. It was reported that LIF could protect neurons by upregulating the superoxide dismutase 3 after ischemic stroke [23]. We concluded that the protective effects of LIF occur during early period following stroke. It is known that various immune cells contribute to inflammation and that these cells can express inflammation-related proteins on their surface or release inflammation-related factors to their extracellular environments [24, 25]. Pro-inflammatory genes accelerate the damage of ischemic tissue while anti-inflammation related genes promote the repair of ischemic tissue. In this study, 87 inflammation-related genes were expressed at all the time points (which includes cytokines and their cognate receptors, complements and their cognate receptors, surface antigens, C-type lectin family, and major histocompatibility complex) and were analyzed for their temporal expression patterns.

Among our identified genes, CD16, CD32, CD86, and CD11b were expressed on the surface of many cell types including some microglia, macrophage, T lymphocytes and B lymphocytes. TNF-α, and IL-1β were released by these inflammatory cells. Many studies demonstrated the up-regulation of the aforementioned related genes that promote the inflammatory response [26-29]. In contrast, CD206, Arg1, and Ym1, among our identified genes, were expressed on the surface of anti-inflammatory cells. IL-10 and TGF-β are released by the anti-inflammatory cells, to promote recovery from ischemic stroke [27, 30-32]. We reported that the expression level of pro-inflammatory genes peaked mostly at D14 whereas the anti-inflammation related genes: Arg1 and Ym1 peaked at D1, IL-10 peaked at D7, TGF-β and CD206 peaked at D7. This indicated that the peak time of expression level of pro-inflammatory related genes was later than anti-inflammation related genes. Previous studies using different stroke models have also reported similar tendencies with pro-inflammatory and anti-inflammatory responses.

However, pro-inflammatory and anti-inflammatory responses following stroke could be more complex considering that these related genes are in different cell types, such as microglia, astrocytes, macrophages, lymphocytes and others. In future investigations, we should attempt to identify the proper time point to disrupt the deleterious inflammatory responses, and study inflammatory responses across the entire region of ischemic tissue in the long-term. The current study has provided new insights and detailed information on the molecular biology and pathology, especially with respect to pro-inflammatory and anti-inflammatory responses of experimental ischemic stroke. These data may be useful in the development of therapeutic interventions for ischemic stroke in humans.

## References

[1] Mozaffarian D, Benjamin EJ, Go AS, Arnett DK, Blaha MJ, Cushman M, et al (2016). Executive Summary: Heart Disease and Stroke Statistics--2016 Update: A Report From the American Heart Association. Circulation, 133: 447-454. 26811276 10.1161/CIR.0000000000000366

[2] Dirnagl U, Iadecola C, Moskowitz MA (1999). Pathobiology of ischaemic stroke: an integrated view. Trends Neurosci, 22: 391-397. 10441299 10.1016/s0166-2236(99)01401-0

[3] Iadecola C, Anrather J (2011). The immunology of stroke: from mechanisms to translation. Nat Med, 17: 796-808. 21738161 10.1038/nm.2399 PMC3137275

[4] Lo EH, Dalkara T, Moskowitz MA (2003). Mechanisms, challenges and opportunities in stroke. Nature reviews. Neurosci, 4: 399-415. 10.1038/nrn1106 12728267

[5] Vidale S, Consoli A, Arnaboldi M, Consoli D (2017). Postischemic Inflammation in Acute Stroke. J Clin Neurol (Seoul, Korea), 13: 1-9. 10.3988/jcn.2017.13.1.1 PMC5242162 28079313

[6] Kim JY, Park J, Chang JY, Kim SH, Lee JE (2016). Inflammation after Ischemic Stroke: The Role of Leukocytes and Glial Cells. Exp Neurobiol, 25:241-51. 27790058 10.5607/en.2016.25.5.241 PMC5081470

[7] Selvaraj UM, Poinsatte K, Torres V, Ortega SB, Stowe AM (2016). Heterogeneity of B Cell Functions in Stroke-Related Risk, Prevention, Injury, and Repair. Neurotherap, 13: 729-747. 10.1007/s13311-016-0460-4 PMC5081124 27492770

[8] Xiong XY, Liu L, Yang QW (2016). Functions and mechanisms of microglia/macrophages in neuroinflammation and neurogenesis after stroke. Prog Neurobiol, 142: 23-44. 27166859 10.1016/j.pneurobio.2016.05.001

[9] Huang M, Wan Y, Mao L, He QW, Xia YP, Li M, et al (2017). Inhibiting the Migration of M1 Microglia at Hyperacute Period Could Improve Outcome of tMCAO Rats. CNS Neurosci & Therap, 23:222-232. 27991729 10.1111/cns.12665 PMC6492671

[10] Meller R, Pearson AN, Hardy JJ, Hall CL, McGuire D, Frankel MR, et al (2016). Blood transcriptome changes after stroke in an African American population. Ann Clin Transl Neurol, 3: 70-81. 26900583 10.1002/acn3.272 PMC4748310

[11] Pereira AC, Gray JD, Kogan JF, Davidson RL, Rubin TG, Okamoto M, et al (2017). Age and Alzheimer’s disease gene expression profiles reversed by the glutamate modulator riluzole. Mol Psychiatry, 22: 296-305. 27021815 10.1038/mp.2016.33 PMC5042881

[12] Chen K, Deng S, Lu H, Zheng Y, Yang G, Kim D, et al (2013). RNA-seq characterization of spinal cord injury transcriptome in acute/subacute phases: a resource for understanding the pathology at the systems level. PloS One, 8: e72567. 23951329 10.1371/journal.pone.0072567 PMC3739761

[13] Li J, Li Y, Ogle M, Zhou X, Song M, Yu SP, et al (2010). DL-3-n-butylphthalide prevents neuronal cell death after focal cerebral ischemia in mice via the JNK pathway. Brain Res, 1359: 216-226. 20800583 10.1016/j.brainres.2010.08.061 PMC3099257

[14] Ramos-Cejudo J, Gutierrez-Fernandez M, Rodriguez-Frutos B, Exposito Alcaide M, Sanchez-Cabo F, Dopazo A, et al (2012). Spatial and temporal gene expression differences in core and periinfarct areas in experimental stroke: a microarray analysis. PloS One, 7: e52121. 23284893 10.1371/journal.pone.0052121 PMC3524135

[15] Morancho A, Rosell A, Garcia-Bonilla L, Montaner J (2010). Metalloproteinase and stroke infarct size: role for anti-inflammatory treatment? Ann N Y Acad Sci, 1207: 123-133. 20955435 10.1111/j.1749-6632.2010.05734.x

[16] Cai H, Mu Z, Jiang Z, Wang Y, Yang GY, Zhang Z (2015). Hypoxia-controlled matrix metalloproteinase-9 hyperexpression promotes behavioral recovery after ischemia. Neurosci Bull, 31: 550-560. 25975730 10.1007/s12264-015-1533-1 PMC5563676

[17] Sezer S, Ucar F, Ulusoy EK, Erdogan S, Bilen S, Zungun C, et al (2014). Serum amyloid A, fetuin-A, and pentraxin-3 levels in patients with ischemic stroke: novel prognostic biomarkers? Turkish J Med Sci, 44: 16-23. 10.3906/sag-1211-90 25558553

[18] Bonaventura A, Liberale L, Vecchie A, Casula M, Carbone F, Dallegri F, et al (2016). Update on Inflammatory Biomarkers and Treatments in Ischemic Stroke. International J Mol Sci, 17:E1967. 10.3390/ijms17121967 PMC5187767 27898011

[19] Pascotini ET, Flores AE, Kegler A, Gabbi P, Bochi GV, Algarve TD, et al (2015). Apoptotic markers and DNA damage are related to late phase of stroke: Involvement of dyslipidemia and inflammation. Physiol Behav, 151: 369-378. 26253215 10.1016/j.physbeh.2015.08.005

[20] Jia J, Guan D, Zhu W, Alkayed NJ, Wang MM, Hua Z, et al (2009). Estrogen inhibits Fas-mediated apoptosis in experimental stroke. Exper Neurol, 215: 48-52. 18950622 10.1016/j.expneurol.2008.09.015 PMC2651740

[21] Xu XR, Carrim N, Neves MA, McKeown T, Stratton TW, Coelho RM, et al (2016). Platelets and platelet adhesion molecules: novel mechanisms of thrombosis and anti-thrombotic therapies. Thrombosis J, 14: 29. 10.1186/s12959-016-0100-6 PMC5056500 27766055

[22] Choi JS, Park J, Suk K, Moon C, Park YK, Han HS (2011). Mild Hypothermia Attenuates Intercellular Adhesion Molecule-1 Induction via Activation of Extracellular Signal-Regulated Kinase-1/2 in a Focal Cerebral Ischemia Model. Stroke Res Treat, 2011: 846716. 10.4061/2011/846716 PMC3118291 21716663

[23] Davis SM, Collier LA, Leonardo CC, Seifert HA, Ajmo CT Jr., Pennypacker KR (2017). Leukemia Inhibitory Factor Protects Neurons from Ischemic Damage via Upregulation of Superoxide Dismutase 3. Mol Neurobiol, 54: 608-622. 26746670 10.1007/s12035-015-9587-2 PMC5026633

[24] Pfluecke C, Berndt K, Wydra S, Tarnowski D, Barthel P, Quick S, et al (2016). Atrial fibrillation is associated with high levels of monocyte-platelet-aggregates and increased CD11b expression in patients with aortic stenosis. Thrombosis Haemostasis, 115: 993-1000. 26763077 10.1160/TH15-06-0477

[25] He Y, Ma X, Li D, Hao J (2016). Thiamet G mediates neuroprotection in experimental stroke by modulating microglia/macrophage polarization and inhibiting NF-kappaB p65 signaling. J Cereb Blood Flow Met, 0:1-14. 10.1177/0271678X16679671 PMC5536801 27864466

[26] Mantani PT, Ljungcrantz I, Andersson L, Alm R, Hedblad B, Bjorkbacka H, et al (2014). Circulating CD40+ and CD86+ B cell subsets demonstrate opposing associations with risk of stroke. Arterioscl Thrombosis Vasc Biol, 34: 211-218. 10.1161/ATVBAHA.113.302667 24202305

[27] Hu X, Li P, Guo Y, Wang H, Leak RK, Chen S, et al (2012). Microglia/macrophage polarization dynamics reveal novel mechanism of injury expansion after focal cerebral ischemia. Stroke, 43: 3063-3070. 22933588 10.1161/STROKEAHA.112.659656

[28] Jeong SI, Shin JA, Cho S, Kim HW, Lee JY, Kang JL, et al (2016). Resveratrol attenuates peripheral and brain inflammation and reduces ischemic brain injury in aged female mice. Neurobiol Aging, 44: 74-84. 27318135 10.1016/j.neurobiolaging.2016.04.007

[29] O’Hare FM, Watson W, O’Neill A, Grant T, Onwuneme C, Donoghue V, et al (2015). Neutrophil and monocyte toll-like receptor 4, CD11b and reactive oxygen intermediates, and neuroimaging outcomes in preterm infants. Pediat Res, 78: 82-90. 25826119 10.1038/pr.2015.66

[30] Shin JA, Lim SM, Jeong SI, Kang JL, Park EM (2014). Noggin improves ischemic brain tissue repair and promotes alternative activation of microglia in mice. Brain, Behav, Immun, 40: 143-154. 24704569 10.1016/j.bbi.2014.03.013

[31] Cuartero MI, Ballesteros I, Moraga A, Nombela F, Vivancos J, Hamilton JA, et al (2013). N2 neutrophils, novel players in brain inflammation after stroke: modulation by the PPARgamma agonist rosiglitazone. Stroke, 44: 3498-3508. 24135932 10.1161/STROKEAHA.113.002470

[32] Li C, Wang J, Fang Y, Liu Y, Chen T, Sun H, et al (2016). Nafamostat mesilate improves function recovery after stroke by inhibiting neuroinflammation in rats. Brain Behav Immun, 56: 230-245. 27033633 10.1016/j.bbi.2016.03.019

